# Influence of School Backpack Load as a Variable Affecting Gait Kinematics among Seven-Year-Old Children

**DOI:** 10.3390/ijerph19073843

**Published:** 2022-03-24

**Authors:** Paulina Tomal, Anna Fryzowicz, Elżbieta Skorupska, Lechosław B. Dworak

**Affiliations:** 1 Department of Physiotherapy, Karol Marcinkowski Poznan University of Medical Sciences, 61-545 Poznan, Poland; skorupska@ump.edu.pl; 2 Department of Biomechanics, Poznan University of Physical Education, 61-871 Poznan, Poland; fryzowicz@awf.poznan.pl; 3 Faculty of Health Sciences, Calisia University, 62-800 Kalisz, Poland; l.dworak@akademiakaliska.edu.pl

**Keywords:** backpack, schoolchildren, gait, kinematic, biomechanics, weight, load carriage

## Abstract

This article investigates schoolchildren’s ability to carry an additional load using a backpack (BP). According to scientific research, there is no precise limit to the maximum backpack load, which varies from 10% to 15% of body weight (BW). The purpose of this study was, therefore, to evaluate the influence of an additional external load carried using a backpack on gait kinematics among seven-year-old children in Poland, including assessment of the gender differences. The study was conducted among 26 (13 boys and 13 girls) primary school children aged seven years. The children walked at their preferred speed, under four conditions: with no load (0% BW) and with 10%, 15% and 20% BW. Spatiotemporal parameters were measured using the 2 m Footscan^®^ platform system and photocell Sectro timing system. The children walked more slowly under an additional load. Their step length and single support time decreased. Their base of support, step time and double support time increased. There was no significant effect on their stride length or gait cycle time. The gait kinematic changes were most evident between 10% BW and greater loading. The results highlight how children’s gait is affected by carrying additional external loads, which should not exceed 10% BW. That limit is appropriate for both genders.

## 1. Introduction

The adoption of the bipedal posture by humans, throughout evolution, has resulted in a change to our way of locomotion. The gait is complex, involving many muscles and based on synchronous neuromuscular coordination of segments of the human body. It is a repetitive sequence of limb movements aimed at shifting the body in a designated direction, ensuring stability in the phase of foot contact with the ground [1]. When the locomotor system performs gait activity, there should be an optimal energy expenditure [2]. 

As a child, carrying an additional external load that affects the upright posture has an impact on the musculoskeletal system’s development, with long-term consequences. When children attempt to maintain their vertical body posture, gravitational forces act on their body segments, forming vertebral column curves in the sagittal plane: cervical, lumbar lordosis and thoracic kyphosis [3]. 

The most comfortable way to transport an additional load is using a backpack (BP). Yet, an additional external load, placed on the back may still affect the head tilt, cervical angle and trunk flexion [4,5,6,7] and potentially increase the lumbar lordosis angle [8,9]. 

Epidemiological studies indicate that, in recent years, children have more often reported pain in the musculoskeletal system. The most common complaint is back pain [10,11,12]. Authors indicate that carrying too heavy a BP can cause pain in the vertebral column [13,14,15,16] or at least more fatigue [17]. In addition, living a sedentary lifestyle may also contribute to back pain occurrence [16]. Children who report back pain in their adolescence are more likely to develop similar ailments in adulthood [18]. The factor that impacts the occurrence of pain in the musculoskeletal system, is the time the child spends carrying a BP each day. A preventive procedure is to provide the use of school lockers, which affects a reduction in the backpack’s weight. 

The way children wear their backpacks is widely discussed. Wearing a BP on two shoulders is associated with less discomfort, reduced feeling of pressure, a sense of greater stability and easier walking. Children should avoid asymmetric BP carrying, especially on one shoulder [12]. The way of carrying the load depends on factors such as the shape and size of the weight, transport time, environment and individual psychophysical conditions. Postural disorders caused by BPs can be worsened by coexisting obesity or excess weight in school-aged children [19]. Typically, children may choose a two-strap BP, one-strap BP, suitcase, satchel or schoolbag with wheels. The type of school bag influences their pain occurrence, discomfort, posture disorders and ergonomics of movement [17,20].

The call for recommendations assumes we can determine values that are appropriate for all children attending school. Nevertheless, the size of the BP should be adjusted individually according to the somatic parameters of each child [21,22]. The time at primary school is important in the context of the child’s growth and entire development, including somatic changes. It is the time when the spine forms and the gait parameters shape up [23]. The gait of children aged six to eight years can be analyzed similarly to that of adults, taking into account ongoing changes and maturation [24].

Wearing the school bag on the back shifts the center of mass upward, which places stress on the conditions of postural stability [25]. School-age children perform a lot of physical activity, such as walking, running and jumping. Therefore, an analysis of the impact of school BPs should be carried out in dynamic conditions. During dynamic activities, such as running, the momentum of the head and torso mass increases [8,9,20].

In Poland, children begin their school education aged seven years, regularly carrying BPs. They regularly pack items and books that are unnecessary on a given day in their BP, leading to excessive loads. To date, there is no evidenced limit on the weight of a school BP that can safely be used by school-aged children. The weight of a school BP varies depending on a child’s age and education level [26,27,28]. 

Scientific recommendations advise that a school BP weight should not exceed 10% or 15% body weight (BW) [29,30]. However, the BP load very often exceeds these recommended values. Plenty of research has investigated the influence of an additional external load on children older than seven years or adults [5,17,27,31,32,33,34,35,36,37]. 

Nevertheless, there is still a need to investigate the influence of an additional external load on factors such as gait, especially during activities. The use of objective and advanced techniques is recommended in research to this end, with consideration of gender differences, as to date, there is no overall agreement about gender differences in gait kinematics [38,39,40]. As there is no evidenced information about the influence of the BP load on spatiotemporal gait parameters, especially for children aged seven years and including the investigation of gender differences, there is a need for research to be conducted, taking advanced measurements to fill the existing research gap. The issue is a relevant one when we consider the high susceptibility of children’s body posture and musculoskeletal system to changes brought by carrying a heavy load. 

The main aim of this study was, therefore, to determine the influence of the variable weight of a school BP when carried by seven-year-old children, depending on the gender. To that end, we tested girls (G) and boys (B) on the values of characteristic temporal and spatial parameters of walking, with loads equal to 10%, 15% and 20% of BW, as investigated according to a methodology that followed safe, approved protocols.

## 2. Materials and Methods

The study sampled 26 children aged seven years: 13 girls and 13 boys (Table 1). The children attended a primary school in Poznan, Poland, which was selected randomly, but with the condition of organizing a “biomechanical locomotion laboratory” there for three months. The population of this city was 545,680 inhabitants. All the children were first-grade students. The inclusion criteria were as follows: no postural disorders, no bone and muscular deformations that affected the gait parameter values, no pain of the gait, no damage to the lower limbs and no neurological dysfunction. Signed informed consent for the child’s participation from the parent or legal guardian, after a detailed briefing about the purpose and nature of the research, was necessary for a child to be included in the research. The presented research project received approval from the Bioethics Committee, as well as the consent of the school management and teachers. 

We compared data on anthropometrics obtained in the research with normative data in the literature. The girls presented higher body masses, heights and BMIs than those in the literature. Boys’ body masses were also higher, but they had similar heights and BMIs to those in the literature [41,42]. There were no significant differences between the boys’ and girls’ anthropometrics data. 

Children were also asked about their everyday activities after school (whether they were involved in sporting activities or they lived a sedentary lifestyle). In all, 14 children (8 girls and 6 boys) answered that they were involved in sporting activities after school such as swimming, football, gymnastics or other outdoor activities, while 12 children (5 girls and 7 boys) said that they lived a sedentary lifestyle, watching TV or playing games after school.

The weights of the children’s school backpacks were measured for five days of the week, from Monday to Friday. A certified electronic medical scale (Radwag WPT 60/150 OW) was used to take these measurements.

By measuring the children’s body weights, the values corresponding to 10%, 15% and 20% of the child’s BW were determined, to determine the potentially appropriate load of the school BP. The appropriate load was simulated accordingly by selecting appropriately weighted books. A school BP design that is commonly available and meets the requirements of ergonomic and modern design (Herlitz, Smart, Berlin, Germany,) was used in the study. The weight of the BP was 290 g. The average value of the applied load (taking into account the mass of the school BP used) was: 1.884 ± 0.73 kg for a 10% BW load, 3.276 ± 1.1 kg for a 15% BW load and 4.668 ± 1.47 kg for a 20% BW load. 

**Figure 1:**
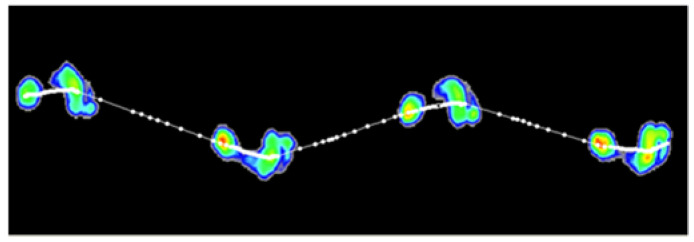
Roll-off screen (Footscan^®^ platform system).

The measurement of the spatiotemporal parameters of the gait was performed using the Footscan^®^ platform system (RSscan International, 2 m × 0.4 m × 0.02 m, with 16,384 sensors). The same measurement track was used in other studies [43,44]. Footscan 7 gait second-generation software was applied in the measurement procedure (Figure 1). The photocell Sectro timing system was used to measure the average walking speed. The measurement technique consists of determining the time between switching on the photocell at the beginning of the measuring path and switching off the next one at its end. The distance between the photocells is fixed. The signal that turns the system on and off is induced by a change in the continuity of the infrared light beam between the transmitter and receiver.

The children walked at their preferred speed on a four-meter-long path, without shoes. Photocells and receivers were located at a height of 70 cm from the ground. The walking speed (v) was calculated as the quotient of the distance traveled (∆S) to the time taken for travel (∆t).

**Figure 2:**
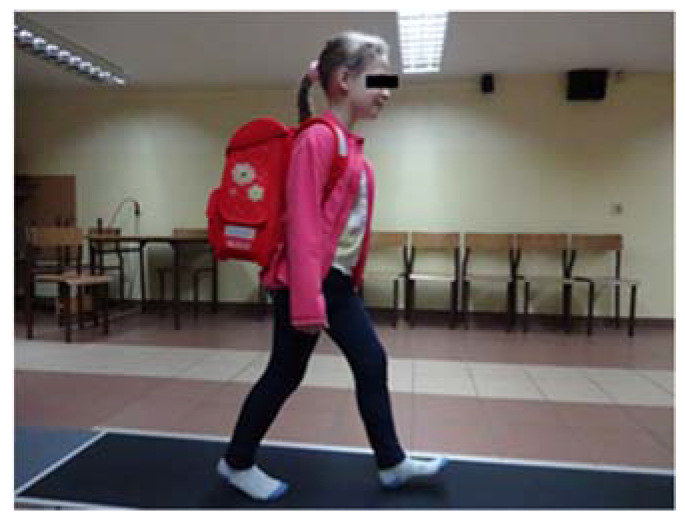
Measurement path including level adjustments and research environment.

Before taking the appropriate measurements, in a pilot experiment, the children were familiarized with the test location (a private, intimate room), platform (dimensions, texture and stiffness of the substrate) and the test method. They made several trial passes. The study included four variable gait measurement conditions: unloaded and, backpacked with a load of 10%, 15% and 20% BW, (Figure 2). The children wore the BP on both shoulders symmetrically.

**Figure 3:**
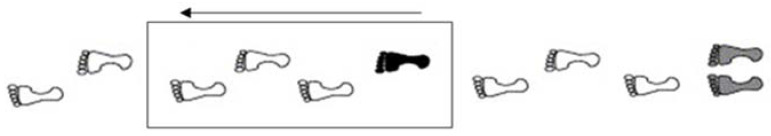
Scheme of the mid-gait protocol (developed based on the available sources).

The statistical analysis of the test results included arithmetic mean values obtained from five correctly performed measurements. The study used the mid-gait protocol (Figure 3), in which it is recommended that the foot contact with the platform should take place at least on the fourth step [45,46].

The following spatiotemporal parameters were analyzed: step length and stride length (cm), heel–heel base of support (cm), step time (ms), gait cycle time (ms), single support time (ms), double support time (ms) and walking speed (m/s). The width of the step (cm) was determined by the distance from the points located in the middle of the right and left heels (h–h base of support). 

Statistical analysis was performed using STATISTICA 10.0 software (StatSoft, Inc., Tulsa, OK, USA). Data were described as the mean ± SD. 

To evaluate differences between the limbs, dependent *t*-test for paired samples or Wilcoxon signed-rank test was applied. To evaluate gender differences, the independent two-sample t-test or Mann–Whitney U test was carried out. All assumptions for conducting ANOVA were checked in advance, including normality, homogeneity and sphericity. Mauchly’s test was used to evaluate the sphericity. Degrees of freedom were corrected in case sphericity’s assumption was violated. Greenhouse–Geisser estimation was applied. The gait data were tested for normality using the Shapiro–Wilk test, set at a significance level of *p* < 0.05. A general linear model with repeated measures was created to compare the differences in the parameters for four measurements. Tukey’s post hoc test was carried out to indicate the differences between measurements with different BP loads. 

The effect size was computed using eta-squared (ŋ^2^). It was defined as without effect if 0 < ŋ^2^ < 0.04, a minimum effect if 0.04 < ŋ^2^ < 0.25, a moderate effect if 0.25 < ŋ^2^ ≤ 0.64 and a strong effect if ŋ^2^ > 0.64. 

## 3. Results

**Figure 4:**
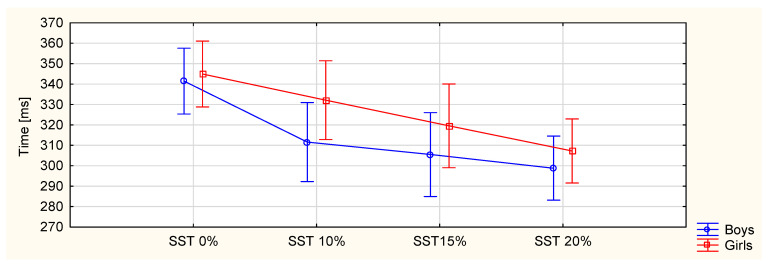
Single-support-time (SST) values for different BP loads.

**Figure 5:**
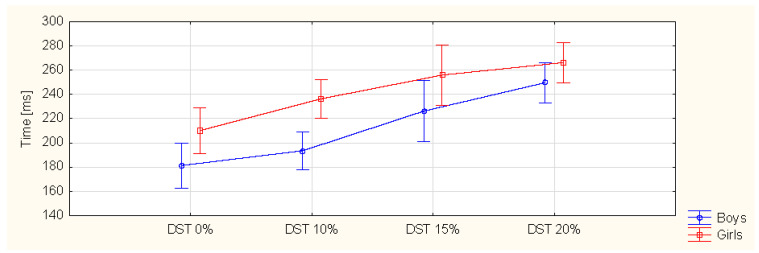
Double-support-time (DST) values for different BP loads.

The results obtained from the research are presented in Table 2 and Figure 4 and Figure 5. 

### 3.1. Limb Differences

There were no differences between the right and left sides in the analyzed data. The arithmetic average of the obtained results for the right and left legs was assessed. 

### 3.2. Spatial Parameters

The step length was less with an additional external load, both for boys (*p* = 0.006) and girls (*p* < 0.001). In the girls’ group, there was a significant difference between 20% BW and all others loads. For boys, there was a difference between 20% and 0 BW%. There was no statistical difference in stride length for either group. 

The h–h base of support increased in both groups with increasing loads (*p* < 0.001). For girls, the statistical difference was between 20% BW and the rest of the measurements. For boys, there was a difference between both 15% and 20% BW versus the others. There were no gender differences in spatial parameters. 

### 3.3. Temporal Parameters

The step-time parameters statistically increased for the boys (*p* < 0.001) and girls (*p* = 0.001). There was a difference between the boys and girls in the 10% BW results (*p* = 0.003). There was also a statistical difference between 20% BW and the other measurements for both boys and girls. There was no statistical difference in the gait cycle time for either group. There were gender differences for 0% BW (*p* = 0.044), 10% BW (*p* = 0.005) and 15% BW (*p* = 0.037). The single support time decreased with an increasing load for both boys and girls (*p* = 0.001), with no gender difference. For boys, there were differences between the measurement with no load (0% BW) and the other measurements. For girls, there were differences between 0% BW and both 15% and 20% BW. There were changes between 10% and 20% BW. The double support time was longer with an increasing load, for both boys and girls (*p* < 0.001). There were gender differences for 0% BW (*p* = 0.033) and 10% BW (*p* = 0.001). For girls, there were changes in the parameters between 0% BW and all three weighted loads. For boys, the changes were between 0% BW and just 15% and 20% BW. There was also a difference between 15% and 20% BW. The velocity decreased with an increasing load (*p* < 0.001) with no gender difference. For girls, the differences were between 0% BW and the three weighted loads and between 20% BW and the other two weighted loads. 

## 4. Discussion

This is the first study to present the spatiotemporal gait parameters under four different loading conditions (0%, 10%, 15% and 20% BW) of additional load carried in the backpack by seven-year-old girls and boys. In contrast to other similar research [47,48], the novelty of our study is that only first-grade students who had just started using BPs, were examined. Therefore, the early effects of using BPs were captured. The most relevant finding of this study is that additional external loads exceeding 10% BW resulted in reduced step length, increased h–h base of support, increased step time, decreased single support time and increased double support time compared to external load of 10% BW or less. 

### 4.1. Spatial Parameters

The spatiotemporal parameters obtained for the children walking with no external load were similar to normative values presented in other works [39,40]. 

The step length decreased with an increasing load in both genders, with no gender effect in the obtained data. The experimental effect was also similar for both groups. In both groups, a statistical difference was found for the 20% BW load. The obtained results are consistent with those presented in other works. Chow et al., for instance, applied loads of 0%, 7.5%, 10%, 12.5% and 15% BW to analyze the gait of 22 girls, and found a statistically significant reduction in their step length at loads above 10% BW [49].

Hong et al. demonstrated a reduction in step length with loads of 15–20% BW compared to a load of 10% BW. It should be noted that the children they studied moved at a constant speed of 1.1 m/s, while in the presented work, they moved at any comfortable speed [50]. Ahmad et al. observed no statistical effect for any of the analyzed spatiotemporal parameters. In their research, they assessed 57 primary school students (seven to nine years old) as they performed four 10 m walks, carrying 0%, 10% of 15% of their body mass in a BP. A floor-based photocell system was used to collect the main spatiotemporal gait kinematics [51]. They suggested that kinetic and physiological changes occurred regardless of kinematic changes to adapt to increasing loads. The physiological interaction with external loads may connect with changes in the cardiorespiratory system and increasing energy expenditure [51,52]. 

A slight decrease in stride length was observed in the girls’ group. However, the changes were not statistically significant. This study partially confirms the results obtained by Pascoe et al., who examined children aged 11–13 years. They observed a decrease in stride length under the influence of symmetric and asymmetric loads of 17.7% BW [4].

Connolly et al. analyzed the gait of 32 children aged 12–13 years and observed a reduction in the stride length with an asymmetric load and an increase in stride length with a symmetric load, though the changes were not statistically significant [53].

Singh et al. observed the stride length of 17 boys aged 9.7 years on average under the influence of three load variants. They did not find a statistically significant difference for the stride length between different load variants (0%, 10%, 15% and 20% BW) [54]. However, a reduction in stride length was observed by Pau et al., who examined 218 children aged 6–13 years. Under the influence of an additional load of 15% BW, this parameter decreased by 10 cm. However, the difference was not statistically significant [55]. Razali et al. examined six girls aged 9–10 years and noted a decrease in their stride length for a school BP load of 15% BW [21]. Studies carried out on adults reported similar findings to those obtained for children. LaFiandra et al. examined a group of 12 people aged 26. The BP load was 40% BW. The subjects walked at various speeds (from 0.01 to 1.6 m/s) on a treadmill integrated into a dynamometric platform. The authors did not find an effect of the BP used on the length of the gait cycle [56].

In this study, the step width increased in the girls’ group by 1.92 cm for the 15% BW load and by 4.66 cm for the 20% BW load. In the boys’ group, the parameter increased by 2.38 cm for the 15% BW load and 4.2 cm for the 20% BW load. This may have been due to compensatory changes related to maintaining their balance.

Connolly et al. found a reduction in the walking width while walking with a BP load of 15% BW (compared to measurement without a load). The value decreased by 0.48 cm for a symmetrically placed BP. However, the observed changes were not statistically significant [53]. Measurements were made of the distance from the heel of one foot to the toe of the other foot. In the presented study, measurements were made based on the distance between the center of the heels.

### 4.2. Temporal Parameters

The step-time parameters increased with increasing loads for both groups. There was a statistical difference between 20% BW and the other conditions for both boys and girls. However, no change in the gait cycle time was observed. Chow et. al. observed decreasing step time with gait cycle time decreases [49]. For the gait cycle time parameter, there were gender differences in most measured conditions, except for 20% BW. 

The single support time decreased for both genders in most of the analyzed conditions. The obtained results were consistent with those presented by Chow et al., who found reduced single support times under external loads. They analyzed the gait of 22 girls with external loads of 0%, 7.5%, 10%, 12.5% and 15% BW [49]. Hong et al. analyzed the gait of the boys aged 10.3 years on average under loads of 0%, 10%, 15% and 20% BW. They showed an increase in the single support time for loads of 15–20% BW [50]. Kinoshita tested 10 adult males using 20% and 40% BW loads. A reduction in the single support time with an increasing external load was reported [57]. The change may be related to the simultaneous extension of the double support time. 

The double support time parameter increased with the external load applied. There were gender differences for the measurements with no load and 10% BW. A statistical effect was observed for girls with 10%, 15% and 20% BW and for boys with 15% and 20% BW. Other authors obtained similar results [49,53,55,57,58].

A statistically significant difference of the double support time was also found in studies by Singh et al. Changes were observed for the 20% BW load when the center of the BP was placed below Th8–9 and for the 10% BW load for a BP above Th8–9 [54]. The increase in the double support time may be related to the influence of the additional load of the school BP on the instability during walking. Increasing the double support time can make gait more stable. Otherwise, instability may result from placing the BP on the back, which increases the height of the COM position, resulting from the weight of the BP and the weight of the child, and reducing the body’s balance. During double support, the position of the COM is lower, which improves the stability of the gait [52,54].

Connolly et al. observed a decrease in gait velocity for measurements with an asymmetric load and an increase in speed with a symmetric load. However, the changes were not statistically significant [53]. Research by other authors confirmed the results obtained in the presented study [27,51]. A significant reduction in gait velocity for a load of 20% BW was found by Singh et al. The authors suggested that a lower walking speed for a BP placed lower on the spine may minimize the energy expenditure [54].

In the presented study, the top of the BP was at the C7-Th1 level. The results we obtained were consistent with those reported in the work of Wang et al., who suggested that the reduced gait velocity due to an additional external load in children is similar to that in untrained adults [59].

Applying a load of 15% BW has been shown to reduce the gait velocity [4,21]. Under an additional external load, such a reduction may be associated with minimizing the center of mass oscillation, to avoid musculoskeletal overload. The observed reduction in gait velocity may also have been due to increased stability as additional weight was transferred. If a person moves at any speed while carrying an additional load, the consequence will be a reduction in walking speed and distance. Walking at a slower velocity may result from compensation associated with the increased energy expenditure needed to carry the extra load [57].

This study has some limitations. First, the study group was relatively small. However, the presented results showed statistically significant changes with increased loads and allowed for presenting a precise advised limit of backpack load. In the future, a study with large groups of first-grade students from different primary schools should be performed to overcome this limitation. Second, children were walking barefoot although they normally wear shoes when carrying their backpacks. This was due to the simultaneous measurement of the foot pressure on the floor, the results of which are yet to be published. Third, although we had information on children’s activities after school, there was no muscle strength measurement, which should be performed in future studies.

## 5. Conclusions

In the presented study, increasing the additional external load was shown to influence the spatiotemporal gait parameters. These changes indicate that increasing the BP load changes the step length, width and time, single and double support times and velocity. There are gender differences between most of the temporal parameters but no difference for spatial data. The analyzed parameters mostly differed with BP loads between 15% BW and lower external loads as well as between 20% BW and lower external loads in both gender groups. Boys and girls at seven years old present similar responses to an additional external load. It seems appropriate to avoid external loads higher than 10% BW as a prophylaxis against postural disorders [23,27].

Performing gait tests in schools seems effective for detecting potential threats that may lead to pain and discomfort associated with carrying an additional load (especially in the face of obesity, foot pathologies and posture defects).

An additional load on the back should be carried according to the principles of ergonomics, thus reducing the risk of negative long-term consequences for the development of the musculoskeletal system.

We propose the need for further monitoring of the characteristic parameters of the gait, taking into account the processes of maturation and development of children, as well as the time and distance that children cover each day while carrying an additional load on the back. We also suggest analyzing the kinetic values of the gait and angular displacements of individual body segments under the influence of the applied load of a school BP. Subsequently, we advise analyzing changes in tension within the basic muscle groups during walking. Comprehensively analyzing changes in the kinetic and kinematic parameters under the influence of an additional load will allow changes in the gait pattern to be identified early on, thus flagging possible pathological changes and enabling appropriate prophylaxis to be implemented.

## Figures and Tables

**Table 1:** Participants’ anthropometrics and BP loads (mean ± 1SD).

Gender	N	Body Mass [kg]	Height [cm]	BMI [kg/m^2^]	BP Load Brought to School [kg]	BP load, %BW [%]
Boys	13	26.44 (±7.5)	126.14 (±7.4)	16.38 (±2.8)	2.08 (±0.3)	7.09 (±3.7)
Girls	13	28.29 (±7.5)	126.2 (±7.3)	17.31 (±3.0)	2.41 (±0.6)	8.42 (±1.7)

**Table 2:** Inferential statistics of the spatiotemporal parameters of the gait in different loading conditions.

		Mean (SD)				F	*p*	ŋ^2^
	Gender	0% BW	10% BW	15% BW	20% BW			
Step length [cm]	G	53.61 (2.84)	49.97 (3.77)	48.97 (3.68)	46.18 (3.02)	31.88	<0.001	0.73
	B	52.92 (3.78)	51.23 (4.13)	48.75 (4.22)	44.90 (3.71)	44.60	0.006	0.79
Stride length [cm]	G	98.70 (8.27)	98.93 (8.28)	95.66 (10.12)	95.42 (8.87)	1.17	0.334	0.09
	B	103.01 (10.85)	102.65 (10.40)	94.53 (15.12)	101.43 (9.94)	2.03	0.512	0.14
H–H Base of support [cm]	G	5.07 (2.15)	5.83 (1.76)	6.99 (2.82)	9.73 (3.14)	13.48	<0.001	0.53
	B	6.48 (2.29)	6.16 (1.56)	8.86 (2.11)	10.68 (2.24)	30.34	<0.001	0.72
Step Time [ms]	G	444.49 (34.14)	473.78 (3.65)	482.27 (67.87)	490.63 (39.96)	4.08	0.001	0.25
	B	427.46 (36.68)	415.09 (46.85)	435.15 (68.29)	493.11 (45.23)	12.79	<0.001	0.52
Gait Cycle Time [ms]	G	926.76 (118.32)	931.20 (87.28)	899.97 (116.11)	901.92 (111.58)	0.67	0.578	0.05
	B	842.89 (78.99)	822.11 (93.15)	791.29 (134.96)	881.83 (147.14)	2.21	0.104	0.16
Single Support Time [ms]	G	344.93 (25.53)	332.12 (25.79)	319.56 (33.10)	307.25 (23.18)	7.43	0.001	0.38
	B	341.47 (30.57)	311.62 (40.19)	305.51 (38.37)	298.84 (30.99)	7.35	0.001	0.38
Double Support Time [ms]	G	210.10 (28.87)	236.30 (24.52)	256.02 (41.01)	266.29 (27.85)	13.05	<0.001	0.52
	B	181.28 (36.04)	193.47 (30.02)	226.29 (46.45)	249.74 (29.79)	17.59	<0.001	0.59
Velocity [m/s]	G	0.91 (0.06)	0.87 (0.07)	0.85 (0.08)	0.76 (0.05)	54.86	<0.001	0.86
	B	0.88 (0.13)	0.88 (0.12)	0.88 (0.09)	0.77 (0.10)	10.78	<0.001	0.57

Boys (B), girls (G).

## Data Availability

The data are not publicly available due to data privacy regulations. The data presented in this study are available on request from the corresponding author.
